# Effects of Wind Waves versus Ship Waves on Tidal Marsh Plants: A Flume Study on Different Life Stages of *Scirpus maritimus*

**DOI:** 10.1371/journal.pone.0118687

**Published:** 2015-03-23

**Authors:** Alexandra Silinski, Maike Heuner, Jonas Schoelynck, Sara Puijalon, Uwe Schröder, Elmar Fuchs, Peter Troch, Tjeerd J. Bouma, Patrick Meire, Stijn Temmerman

**Affiliations:** 1 Ecosystem Management Research Group, Department of Biology, University of Antwerp, Wilrijk (Antwerp), Belgium; 2 Federal Institute of Hydrology, Koblenz, Germany; 3 UMR 5023 LEHNA, Université Lyon 1, CNRS, ENTPE, Villeurbanne, France; 4 Department of Civil Engineering, Ghent University, Zwijnaarde (Ghent), Belgium; 5 Royal Netherlands Institute for Sea Research, Yerseke, The Netherlands; Centro de Investigacion Cientifica y Educacion Superior de Ensenada, MEXICO

## Abstract

Recent research indicates that many ecosystems, including intertidal marshes, follow the alternative stable states theory. This theory implies that thresholds of environmental factors can mark a limit between two opposing stable ecosystem states, e.g. vegetated marshes and bare mudflats. While elevation relative to mean sea level is considered as the overall threshold condition for colonization of mudflats by vegetation, little is known about the individual driving mechanisms, in particular the impact of waves, and more specifically of wave period. We studied the impact of different wave regimes on plants in a full scale flume experiment. Seedlings and adult shoots of the pioneer *Scirpus maritimus* were subjected to two wave periods at two water levels. Drag forces acting on, and sediment scouring occurring around the plants were quantified, as these are the two main mechanisms determining plant establishment and survival. Depending on life stage, two distinct survival strategies emerge: seedlings present a stress avoidance strategy by being extremely flexible, thus limiting the drag forces and thereby the risk of breaking. Adult shoots present a stress tolerance strategy by having stiffer stems, which gives them a higher resistance to breaking. These strategies work well under natural, short period wind wave conditions. For long period waves, however, caused e.g. by ships, these survival strategies have a high chance to fail as the flexibility of seedlings and stiffness of adults lead to plant tissue failure and extreme drag forces respectively. This results in both cases in strongly bent plant stems, potentially limiting their survival.

## Introduction

Intertidal marshes provide many important ecosystem services. Apart from ecological functions such as carbon sequestration, water quality regulation, contribution to fishery and providing irreplaceable habitats for specialized organisms [1] they are also important for flood and wave attenuation [2–4] which is why they play a crucial role in coastal defence [5]. However, due to natural disturbances such as storms on the one hand, and direct or indirect human impacts such as land reclamation or human induced sea level rise on the other hand, areas in which marshes can survive or expand naturally are increasingly threatened [6]. A 50% worldwide loss or degradation of salt marshes has occurred over the last two to three decades [7], mainly due to competing economical land use forms for which marshes are embanked and thus lost.

In recent years there has been increasing evidence that seaward marsh expansion by colonization of marsh vegetation onto bare mudflats can be explained by the alternative stable states theory [8–13]. According to this theory, relatively rapid shifts from one stable state to another one (here low-lying bare mudflat to high-elevated vegetated marsh or vice versa) will occur when a critical threshold condition is exceeded, while intermediate states are only transient and unstable. The driving mechanisms for the alternative stable state behaviour in tidal flats and marshes are the local positive feedbacks between sediment elevation and vegetation growth. When—as a result of sedimentation—a tidal flat reaches a threshold elevation where vegetation can establish, this vegetation will subsequently reduce waves and currents and thereby accelerate sedimentation and increase elevation. This will in turn further stimulate vegetation growth leading to enhanced sedimentation etc. until a high vegetated marsh state is reached [14,15].

Friess *et al*. (2012) point out that shifts from bare to vegetated states of intertidal flats depend on critical thresholds that need to be passed. Critical thresholds, meaning “a limit beyond which a state change is ensured” [10], can be for example the biomass of plants as it has been stated that seedlings have to reach a threshold biomass enabling their survival on an intertidal flat [10,16,17]. Elevation relative to mean sea level has been suggested as an important threshold condition for intertidal vegetation establishment based on field data [9] and in various models [12,18,19]. However, elevation only affects vegetation growth indirectly because it lumps the effects of several more directly affecting variables including tidal inundation depth and duration, hydrodynamic forces from tidal currents and waves, and sediment bed dynamics, which are typically correlated to elevation relative to mean sea level. The role of such direct mechanisms in determining threshold conditions for vegetation establishment are only starting to be elucidated by modelling and experimental studies: models indicate that critical thresholds in hydrodynamic conditions, i.e. currents and waves, can determine whether marshes laterally expand or whether marshes erode [2,8,20]. Experimental work has demonstrated that critical thresholds exist in sediment erosion rates (driven by currents and waves) beyond which established vegetation is uprooted [16]. Successful establishment, i.e. a sudden regime shift from bare to vegetated states, occurs when external forcing does not exceed a critical threshold for a certain duration. The mainly species dependent minimum duration of non-disturbance has been defined as “window of opportunity” and their occurrence in disturbance-driven ecosystems such as intertidal marshes depends on the stochastic variability in external forcing, e.g. on a windless period just after seed or rhizome dispersal [15,16].

When looking into the limiting hydrodynamic forces acting on individual plants and marsh edges, waves may be expected to cause critical threshold conditions for pioneer marsh establishment. Waves in estuaries are either wind or ship generated. As estuaries usually represent fetch-restricted environments, ship-generated waves can have an important contribution to the overall wave climate [21,22]. Wind waves in estuaries have short wave periods of 1–2 s, but can be of long duration (hours to weeks). Ship waves on the other hand will reach the shores as outstanding events with one long period primary wave, followed by a train of shorter secondary waves. Their characteristics depend among others on relative speed, load and direction of travel of the ship as well as on local bathymetry [23]: while Curtiss, Osborne, & Horner-Devine (2009) found vessel wake periods of 3–6 s for car ferries for their study site, Houser (2010) reports a range of wave periods of 6 to 17 s. Even longer primary wave periods of up to 90 s, generated by container-vessels, have been observed on the shores of the Scheldt Estuary. The duration of such a ship-generated wave event, i.e. primary and subsequent secondary waves, is in the order of several minutes.

Potential consequences of incoming waves for pioneer plants are (i) drag forces which the plants will have to resist and that will affect the above ground plant material [24,25] and (ii) sediment scouring around the stems, which can in the worst case lead to uprooting [10,26]. These are the two prime mechanisms that have been recognized to cause plant failure or to limit seedling establishment [16,26]. Plants can present adaptation to their environment reducing damage risk by following either an avoidance or a tolerance strategy [27]. Plants will, for example, either avoid drag forces by being highly flexible which allows them to reconfigure under incoming waves, or they will tolerate the forces by having very strong stems highly resistant to breakage.

These wave-induced impacts on plants have typically been linked to parameters as wave height or flow velocities. To our knowledge, no experimental study has so far quantified the effect of different wave periods on pioneer marsh vegetation. The question of wave period becomes highly important when looking to intertidal marshes that are affected by heavy shipping due to the presence of a port. The Elbe Estuary (NW Germany) and the Scheldt Estuary (SW Netherlands) are good examples as they harbour the second and third largest ports of Europe with a shipping traffic of around 600 passages per week through the estuaries. The intertidal marshes that lie downstream of the harbours are thus regularly affected by the high energy long period primary ship wave events accompanying each ship passage.

In a full scale wave flume experiment conducted with seedlings and adult shoots of a plant species colonizing these systems, *Scirpus maritimus*, we addressed the following research questions:

- How does life stage of plants (adult shoots vs. seedlings) influence the resistance of individual plants to wave impact?
- What is the importance of water level at wave impact?
- How does the wave period—natural wind-generated waves (short waves) vs. ship-generated primary waves (long waves)—influence the survival chances of individual seedlings and adult plants?

## Material and Methods

Field access and permission for extraction of plants and collection of seeds from the brackish marshes in the Scheldt Estuary (51°21’47 N, 4°14’53 E) was granted by Natuurpunt (Belgium). We confirm that the experiments did not involve endangered or protected species.

### Wave flume and experimental design

**Fig 1:**
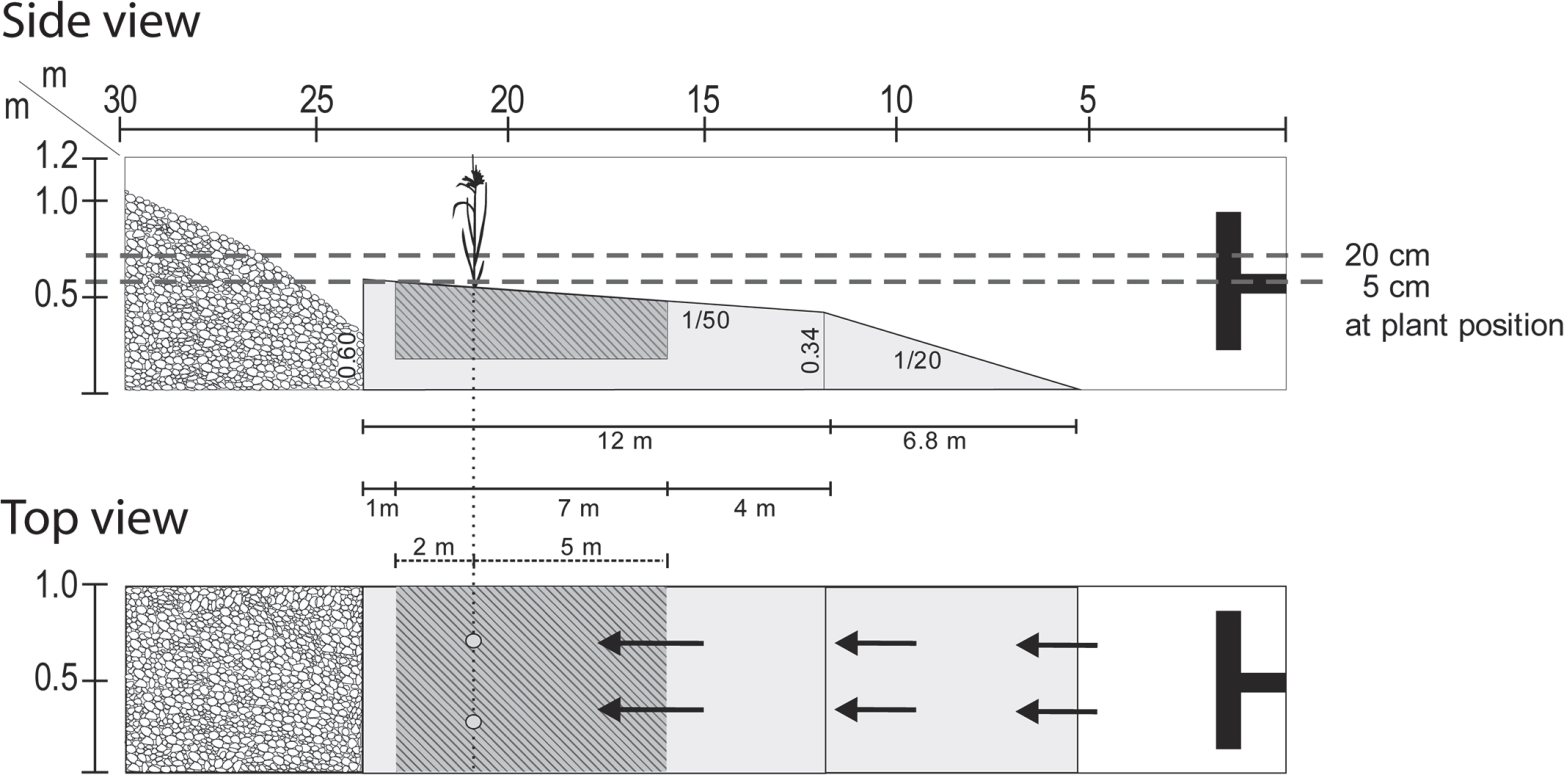
Sketch of physical model in the wave flume. Top: side view; bottom: top view. The position of plants is indicated by the schematic plant on the top panel and by two light grey circles in the bottom panel. The light grey bodies are the 1/20 transition slope and the 1/50 slope of the test section which were built of smooth concrete plates. The sediment box (hatched part of the test section) was filled with natural sediments from the Scheldt Estuary (SW Netherlands). The absorption beach (left end of the flume) was built of pebble stones. The horizontal grey dashed lines (top) represent the two tested water levels (20 and 5 cm water depth at plant position). The black arrows (bottom) represent the direction of propagation of waves produced at the wave paddle (black structure on the right end of the flume).

The experiments were conducted in the wave flume facility at the Department of Civil Engineering at Ghent University (Belgium). The wave tank has a length of 30 m, a width of 1 m and a height of 1.2 m. The physical model (Fig. 1) consisted of a transition slope of 1/20 of 6.8 m length which led to the actual test section with a slope of 1/50 over 12 m. The latter slope is representative for natural marsh-mudflat transition zones in *S*. *maritimus* dominated pioneer vegetation in the Scheldt Estuary (Belgium, SW Netherlands). A section of 7 m of this gentle slope was occupied by a sand box of 0.3 m depth and filled with sand from the Scheldt Estuary (D_50_ = 0.32 mm). A pebble stone absorption beach was placed and maintained at the rear end of the flume in order to avoid wave reflection into the test section.

For each test run, the surface of the sand slope was first brought back into the initial slope of 1/50. Two plants—either two adults or two seedlings—were then transplanted next to each other into the sediment box at marked locations. Wave height at the paddle was set to 17 cm but due to transformation on the slope preceding the plants, the actual wave heights at the plants differed from one hydrodynamic condition to the other (see Table 1). Two wave periods were tested: a 2 s wave period as proxy for natural estuarine near shore wind waves [28], and a 10 s wave period as an artificially generated long period wave, mimicking primary ship waves. A longer wave period was not possible due to technical paddle limitations. Although 10 s waves might not be fully representative for the ship-induced waves observed along the Scheldt Estuary, the comparison of 2 s and 10 s waves in the flume enables us to assess the impact of wave period on the plants. Additionally, two water levels were chosen (5 cm and 20 cm relative to the location of the plants) in order to simulate wave impact at different moments in the tidal cycle or, alternatively, different elevation on the mudflat. Altogether we had four different hydrodynamic conditions (combination of two wave periods and two water levels) for which the two life stages were tested. The test runs consisted of 200 waves each and were repeated five times per condition, i.e. with ten plant replicas for each of the conditions: we considered each plant to be a true replicate as the potential mutual influence of two adjacent plants tested simultaneously can be neglected compared to the forces acting on them from the respective test conditions. Actual wave heights at the paddle and on the test section with plants were measured for each of the tests with resistance wave gauges (sampling frequency 40 Hz). Significant wave heights (H_1/3_) were calculated (Table 1).

**Table 1:** Details of the four tested wave conditions.

Water level (cm)	Wave period (s)	Significant wave height (cm)	
at plants		at paddle	at plants
5	2	17	4
5	10	17	7
20	2	17	11
20	10	17	20

Measured significant wave heights (H_1/3_) are reported.

### Plant material

Forty adult shoots and 40 seedlings of *S*. *maritimus* were used in this experiment. The adult shoots were collected from the brackish marshes in the Scheldt Estuary (51°21’47 N, 4°14’53 E) in April 2012 at the beginning of the growing season. The seedlings were grown from seeds that had been collected in September 2010 at the same location and that had been stored in dry, dark and cool conditions. The plant material was transplanted into PVC tubes of 25 cm height and 12 cm diameter that were lined with plastic bags and filled with the same natural sediment that was used in the flume. Adults and seedlings were grown under equal natural outdoor conditions close to the Scheldt Estuary. They were watered with brackish water (5 g NaCl L^−1^) representative of their natural habitat until they were brought to the flume, where the experiments started end of June 2012.

Our pot-system enabled us to transplant the plants into the flume with their anchored root system contained in the plastic bags without creating any edge effects: this was achieved by removing the bags from the PVC tubes and inserting them into the sediment of the flume where the bags were then folded downward and buried. We then measured the height of the stems and the stem diameter at 3 cm above the sediment. In a later step, plant material from our flume experiment was sampled and analysed for biomechanical traits ([27], see below) in order to better understand the different behaviours of the two life stages in the different hydrodynamic conditions.

### Biomechanical plant traits

We measured biomechanical traits through tensile and bending tests on 20 replicas for each life stage using a universal testing machine (Instron 5942, Canton, MA, USA). For each test, the stem fragments were 10 cm long for adult plants and 5 cm for seedlings. For each sample, we measured the dimensions of the cross section using a digital calliper (± 0.02 mm) at three different points along the sample.

#### Tensile tests

The sample was clamped into the jaws of the machine and a constant extension rate of 5 mm min^−1^ was applied until it broke. We then calculated:

- the *breaking force* (in N) which is defined as the maximum force that the sample can bear without suffering mechanical failure;
- the *tensile strength* (in N m^−2^) which is calculated as the breaking force per cross-sectional area.

#### Bending tests

We performed 3-point bending tests, consisting of a force applied at a constant rate of 10 mm min^−1^ to the midpoint of a sample. We then derived [29]:

- the *Young’s modulus* (*E* in Pa) which quantifies the sample stiffness;
- the *second moment of area* (*I* in m^4^) which quantifies the distribution of material around the axis of bending;
- the *flexural stiffness* (*EI* in Nm^2^) which quantifies the stiffness of the fragment and was calculated by multiplying the obtained *E* and *I*.

### Self-scour

In order to measure the scouring depth, surface and volume produced around the plant stems, we cut off the plant stems close to the sediment bed after each test. The sediment surface was then scanned with a laser scanner (EProfiler from Hydraulic & Coastal Engineering Group, Aalborg University, Denmark) with a horizontal resolution of 5 mm x 5 mm and elevation precision of 1 mm. The scans covered a surface of 0.2 m x 0.2 m around each stem. Reference surfaces next to the plants, hence outside the influence of the stems, were also scanned after each test in order to determine the general deformation of the sediment bed without interference with the plants. Five scans of the initial slope that was flattened to a 1/50 profile before each new test run served for comparison of sheet erosion, i.e. overall sediment erosion independent of local scour.

These data were imported into ArcMap 10.1 (ESRI ArcGIS) where we quantified the scour around each plant. We corrected the scour extent for general slope deformation and wave ripples that modified the slope surface without interference of stems. This was done based on the reference surfaces mentioned above. Calculations on depth, surface and volume were carried out on the corrected data, where we considered the 95-percentile for the maximum scour depth. Furthermore, sheet erosion, i.e. overall erosion of the sediment bed, could be observed and needed to be considered as it might have limited the comparability of the different conditions. In order to determine this type of erosion we compared the reference surfaces resulting from the different wave treatments with the average elevation of the same pieces of the initial slope scans.

### Drag force

We measured drag force on plants under the different hydraulic conditions by attaching the basal part of the cut off plants to strain gauges, calibrated for measurements in N, which were then planted into the sediment bed at the same location where the plants had initially been. The respective test conditions were then run on these replanted plants and the experienced drag forces measured during approximately 2 minutes. Afterwards, we extracted peak drag forces, which coincide with the passage of wave crests, with a LabView program and averaged them for each of the plants. We also considered drag force per frontal area of plants in order to correct the measured forces for the size differences of adults and seedlings. Frontal area was constructed based on our plant property measurements and respective effective water levels (water level + significant wave amplitude) for each of the tests.

### Bending angle

**Fig 2:**
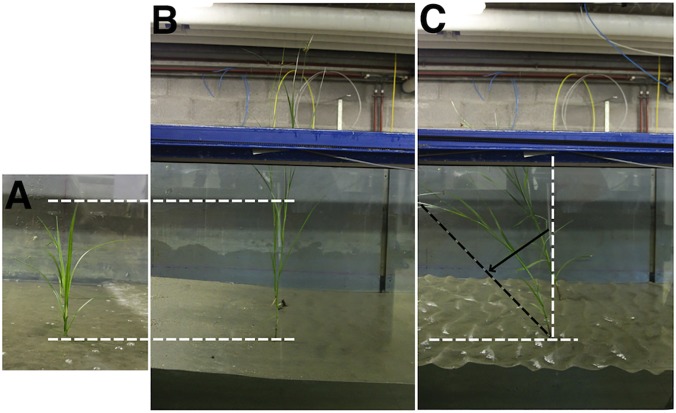
Plant material and definition of bending angle. Seedling **(A)** and adult shoot **(B)** of *S*. *maritimus* before the wave impact. The horizontal dashed lines indicate the size of the seedling relative to the adult. **(C)** illustrates how the bending angle (indicated by the black arrow) of the plants was calculated. Vertical white dashed line: stem position before the test; black dashed line: bending of stem after the test.

The angle of the plant stem with the sediment bed was measured before and after each test and lateral photographs were taken (Fig. 2). Based on these measurements the wave-induced change of the stem angle could be calculated (Fig. 2C). The final bending angles are considered as an indicator for overall plant survival or failure as higher bending angles indicate higher damage to the plant material and less potential for long-term survival. This is founded on the indication that toppling limits survival chances of seedlings [30,31], and that bending can be considered as the mechanism leading to toppling. Field observations from a field transplantation experiment with *S*. *maritimus* shoots and seedlings (publication in prep.) confirm these assumptions. The more a plant is bent, the closer it is to final toppling, i.e. a bending angle of 90°. Furthermore, once the plants lie flat on the sediment bed, they are likely to be covered by sediment, meaning that possibility for photosynthesis will be diminished. Also, the above-ground plant material might start to decompose due to permanent contact with the moist sediment surface and its exposition to benthic activities.

### Statistical analysis

All results have been tested in R with ANOVAs followed by a post-hoc Tukey’s HSD. For the plant properties, i.e. for plant size, stem diameter and biomechanical properties, one-way ANOVAs (parameter ~ life stage) have been considered. For all other flume related analyses, i.e. scour, drag force and bending angle, three-way ANOVAs (parameter ~ life stage*water level*wave period) have been used. In regards to significance, only comparable conditions, e.g. between life stages under equal conditions, or within one life stage for conditions of equal water level or wave period, have been considered as it is not relevant to compare, for example, adult shoots under short wave periods with seedlings under long wave periods.

## Results

### Plant properties

**Fig 3:**
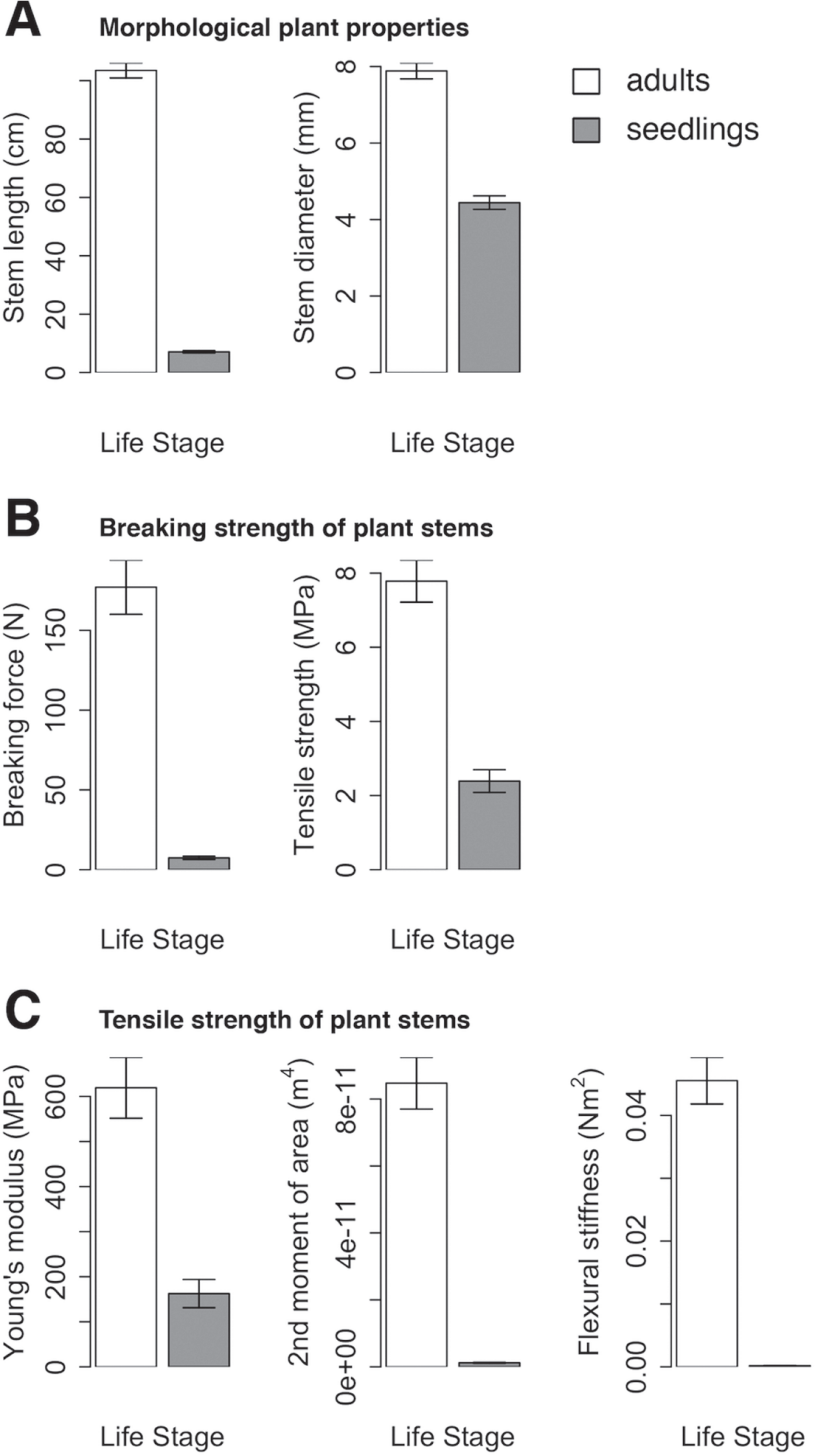
Averages and SE of measured plant traits. **A:** Morphological plant properties as measured at the moment of transplantation to the flume on 40 adults and 40 seedlings; **B** & **C:** Biomechanical traits of stem tissue (**B:** Breaking strength; **C:** Tensile strength) as determined on 20 stems per life stage.

At the moment of transplantation into the flume, the average stem length and diameter of seedlings was significantly smaller than for adults (Fig. 3A) (P < 0.001). The measurements on tensile and bending strength show that adults are significantly stiffer than seedlings as well as significantly more resistant to tensile stress (P < 0.001 for all cases) (Fig. 3B & C).

### Self-scour

**Fig 4:**
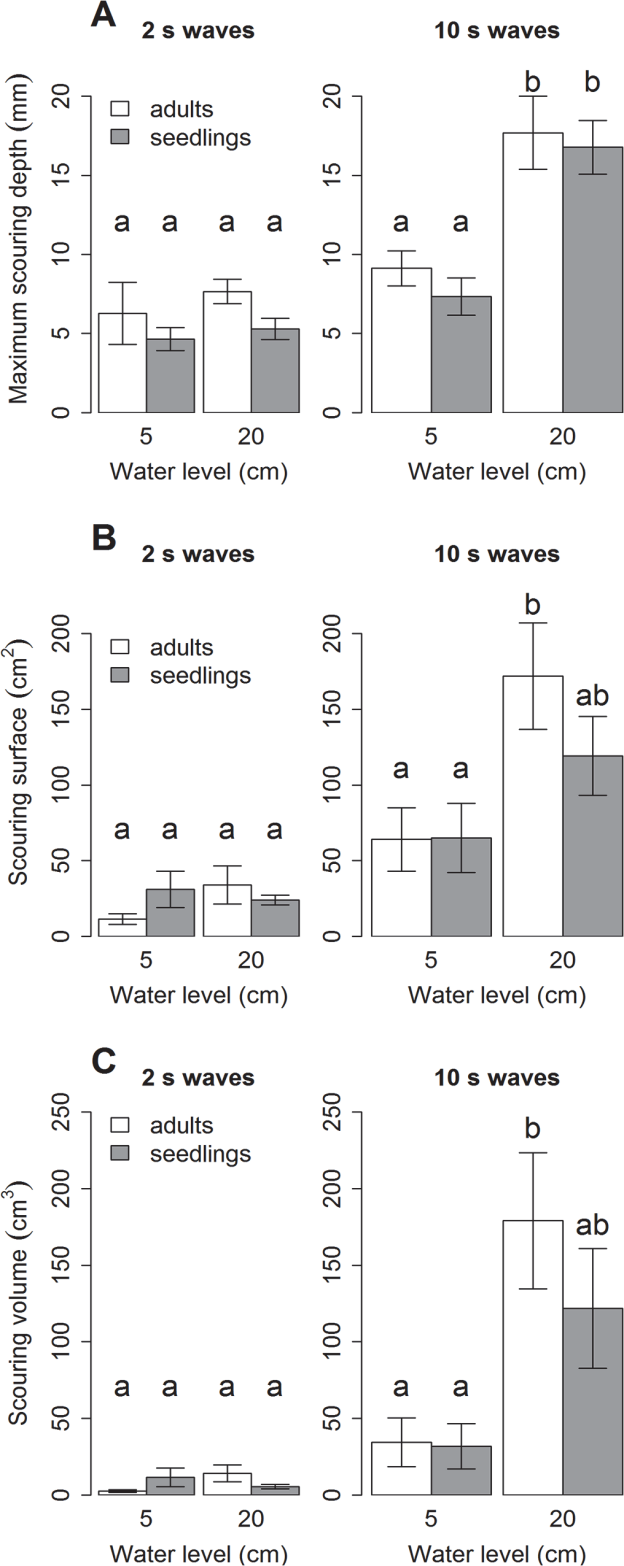
Averages and SE of measured self-scour around plant stems. **A:** Maximum scouring depth (mm, 95-percentile); **B:** Scouring surface (cm^2^); **C:** Scouring volume (cm^3^). Significant differences as resulting from a three-way ANOVA followed by a post-hoc Tukey’s HSD are indicated by different letters.

A three-way ANOVA showed that there were no significant differences in sheet erosion between experiments with different life stages, water levels and wave periods nor between all possible combinations of these conditions which means that results of self-scour can be compared for all conditions. Results on scouring depth (95-percentile), surface and volume all show the same patterns (Fig. 4): there is no significant difference between life stages. Notably, only one condition (water level-wave period combination = 20cm-10s) shows significantly more scouring depth, volume and surface for both life stages.

### Drag force

**Fig 5:**
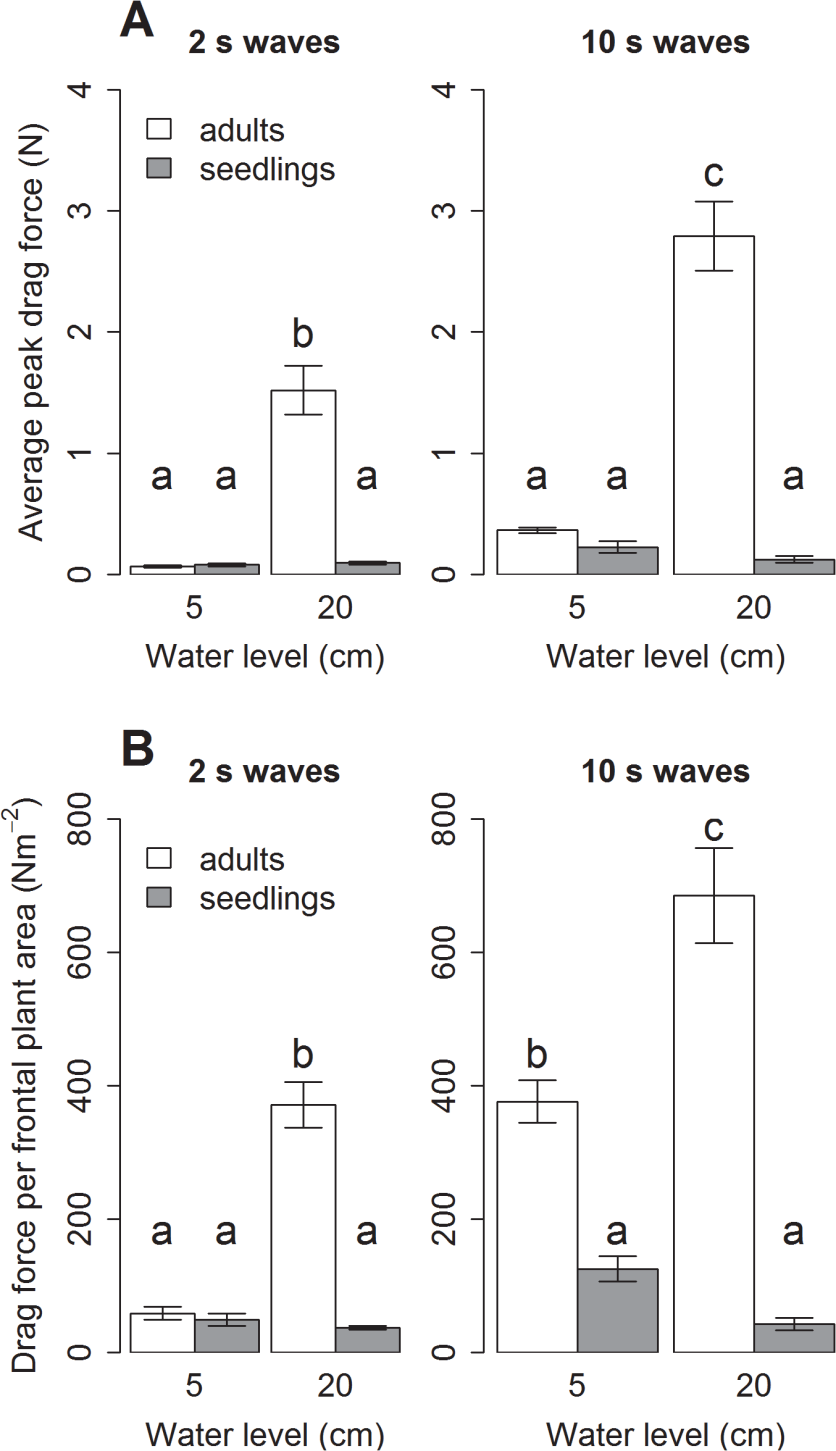
Averages and SE of measured drag forces experienced by plants. **A:** Average peak drag forces (N); **B:** Drag forces per frontal plant surface area (Nm^−2^). Significant differences as resulting from a three-way ANOVA followed by a post-hoc Tukey’s HSD are indicated by different letters.

Results of peak drag forces (Fig. 5A) show that seedlings experience very little drag force under all conditions (< 0.25 N, P = 1 for all combinations of water level and wave period). Adults on the other hand experience significantly more drag force than seedlings at the high water conditions (P < 0.001) for which adults are affected by significantly more drag force from the 10 s waves than from the 2 s waves (P < 0.001). In regards to drag forces per frontal surface area (Fig. 5B) where the size differences between adults and seedlings are considered (Fig. 3A), similar patterns as for average peak drag forces can be observed, except that adults experience significantly more drag force than seedlings for the 5cm-10s condition.

### Bending angle

**Fig 6:**
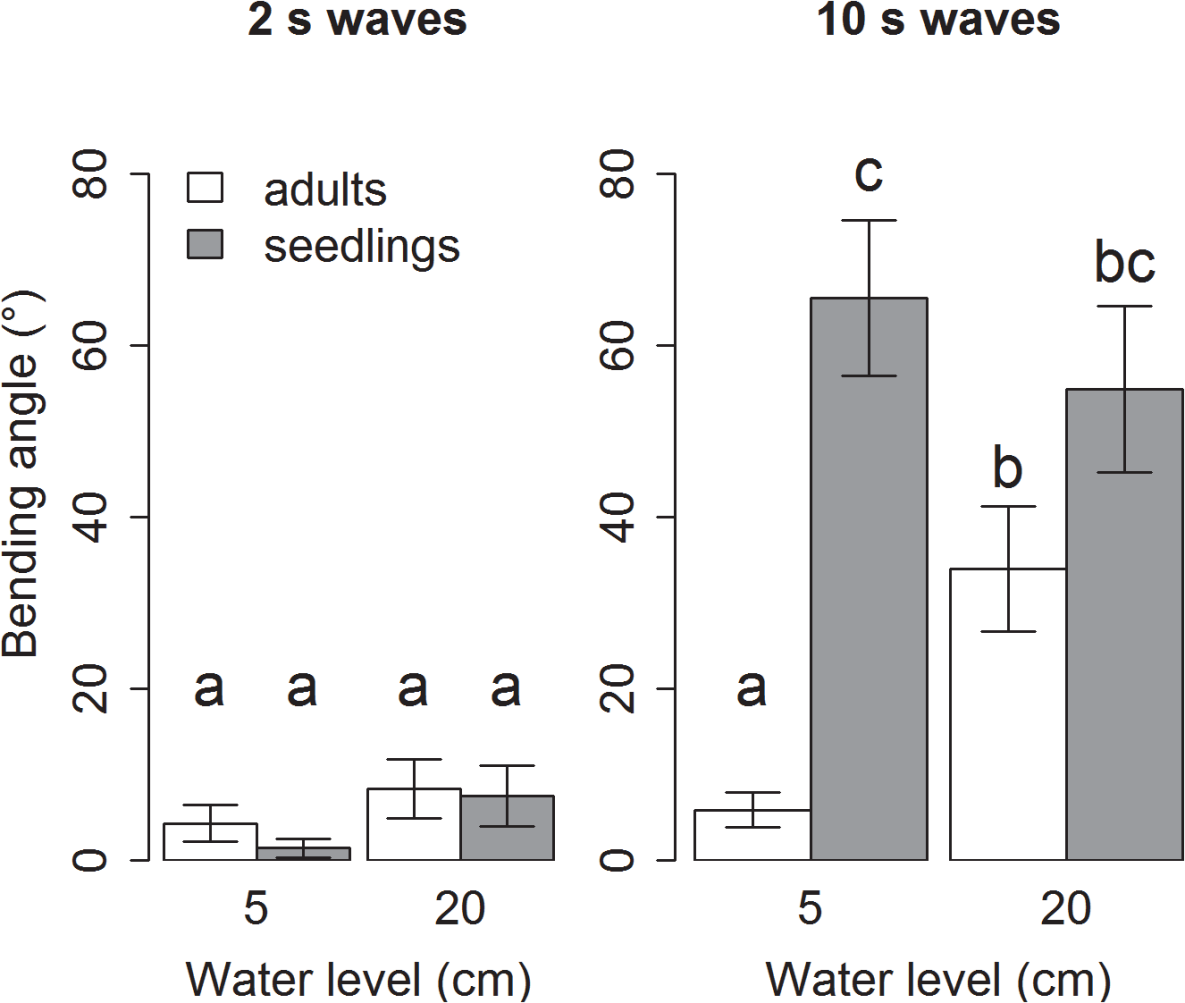
Averages and SE of measured final bending angle of plant stems (°). Significant differences as resulting from a three-way ANOVA followed by a post-hoc Tukey’s HSD are indicated by different letters.

The bending angles of the stems which we measured after the respective tests demonstrate that the 2 s wave conditions at both water levels do not lead to any severe bending (angles of less than 10° on average) for any of the two life stages (Fig. 6). However, when we look at the bending angles for the long wave period, we see that especially the seedlings are strongly affected with bending angles of more than 60° on average at the low water condition and of more than 50° on average at the high water condition. The adults have small bending angles at low water (less than 10° on average) but suffer increasingly at high water (more than 30° on average). Significant differences between life stages for equal conditions only occur for the 5cm-10s condition (P < 0.001). Within one life stage, significant differences occur when the wave period is increased at equal water level (seedlings: 5cm-2s to 5cm-10s, P < 0.001; seedlings and adults: 20cm-2s to 20cm-10s, P < 0.001 and P = 0.03 respectively). For the long wave period, the adults also show significantly more bending at the higher water level as compared to the low water level (5cm-10s to 20cm-10s, P = 0.01).

## Discussion

Elevation relative to mean sea level is generally considered as an indirect lumped threshold variable for shifts from bare to vegetated states of intertidal flats [8–13]. However the role of direct mechanisms that limit or allow vegetation establishment are only starting to be elucidated by experimental studies [32–34]. Here we focused on drag forces, sediment scouring and bending that individual plants experience under wave exposure, showing that both wave period of incoming waves and life stages of plants can act as thresholds for individual marsh plant establishment on the bare mudflat. While the final plant bending angle and experienced drag forces are clearly period and life stage related, no significant differences in self-scour could be observed.

Our results demonstrate that ship-generated waves, mimicked by 10 s waves in our experiment, cause severe bending of the plants while wind waves, mimicked by 2 s waves in our experiment, do not cause any significant bending, neither to the seedlings, nor to the adults, and at neither of the two water levels (Fig. 6). The ship waves lead to strong bending of both adults and seedlings at the higher water level, but at the low water level, only the seedlings are affected. This implies that life stage and water level, although not relevant for wind waves, becomes a critical threshold when exposed to ship waves.

Regarding self-scour, it was found both in engineering literature [35] as well as in ecological plant studies [14,26] that the wave- or flow-induced scour increases with the basal diameter of the obstacle or of the stem in case of plants. Interestingly, our results do not confirm this expectation as the scour is not significantly different for any of the conditions between the two life stages (Fig. 4), although the stem diameters of adults are significantly thicker than the ones of seedlings (Fig. 3A). However, it is possible that the scouring of the seedlings is increased by two parameters: (i) seedlings are more flexible than adults (see Fig. 3C) which leads to bigger movements of the basal parts of the plants when they reconfigure under the incoming waves which could induce additional scour; (ii) the lowest leaves of a seedling are closer to the sediment bed than the ones of adults where only the stem interacts with the sediment (Fig. 2). Overall, the flexible stems of the seedlings and their leaves together might act as a larger obstacle, which results in more scouring than expected from their stem diameter alone.

In terms of drag force, adults experience higher forces than seedlings for the higher water level (Fig. 5A) also when corrected for frontal surface area of the plants (Fig. 5B). This shows that it is not plant surface but plant stiffness that determines the experienced drag (Fig. 3C): flexible plants such as the seedlings experience less drag forces because they can reconfigure depending on external forcing. Reconfiguration has been demonstrated to be one of the traits plants may develop as avoidance strategy of mechanical stresses [27,34,36–39]. Furthermore, the plant tissue and stem diameters of adult plants provide high breaking forces which implies a tolerance strategy (Fig. 3C). In the case of freshwater aquatic plants, a trade-off arrangement where both strategies are negatively correlated has been suggested [27]. Interestingly, we found indications for avoidance versus tolerance strategies within one species between life stages, while the previous studies compare different species [27,36,39]: adults, who have a tolerance strategy, experience high drag forces because of their stiffness, but under the natural wind wave conditions their strategy may protect from suffering mechanical damage. Seedlings, on the other hand, have an avoidance strategy by being extremely flexible and thus by reducing the drag forces through reconfiguration.

Potential limitations of our experiment, putting our findings into perspective, are related to how well natural conditions were reproduced in the lab experiment, both in terms of the used plant material and the simulated wave conditions. For example, adaptation of plants to non-lethal stresses has been observed in experiments where plants have been grown under stressful conditions [31]. Contrary to plants grown in the natural ecosystem, where plant morphology and tissue properties might have adapted to such non-lethal stresses by waves and currents, the plants for this experiment have been grown in sheltered conditions and might therefore have responded differently to the wave induced stresses during the experiments than field plants would have. Another potential limitation is that we applied 200 waves in the lab experiments. While wind waves can indeed reach the shores continuously and thus act on plants and sediments as long as the water level is high enough, long-period primary ship waves only occur at the passage of ships. However, 200 waves do represent the forcing that may be experienced in one week on an estuary with intensive shipping traffic due to an international port such as in the Scheldt Estuary.

This study highlights the importance of wave period for the colonization of bare mudflats by individual seedlings or rhizomes. Adults and seedlings of *S*. *maritimus* appear to be relatively well adapted through their respective package of plant properties for surviving and establishing under natural wind wave conditions at shallow and higher water levels. However, as soon as ship generated, long period waves are present, both survival strategies reach their limits, especially for seedlings, which have a lower chance of survival as strong plant bending is observed even at the lowest water level. This implies that even at the highest parts of the mudflat, seedling establishment may be limited if long period waves interfere. From a broader, evolutionary perspective, our findings underline the fact that the plants and their survival strategies have developed under natural wind wave conditions which is why they are best adapted to resist their impact. This can also be applied to other plant species which either follow the tolerance or avoidance strategy. As their properties have not been adapted to the passage of ships, marsh plants may be less prepared to sustain this kind of stress and their respective wind wave based survival strategy will fail. In the same way, the required window of opportunity for marsh restoration and colonization of the mudflat by individual shoots or seedlings will be hard to achieve: while favourable stochastic deviations from natural perturbations (e.g. windless days) do occur and thus would give a chance for the marsh plants to expand onto the mudflat, the passage of ships cause a more regular distribution of disturbance and therefore a window of opportunity that is long enough for establishment is less likely to occur in the presence of regular ship traffic.

Our results suggest that the establishment of individual shoots and especially seedlings on mudflats may be limited in the presence of long period waves such as typically produced by ships. In estuaries where the management aims at preservation, restoration or expansion of intertidal marshes, this potential impact of ship traffic should be taken into account by taking measures, e.g. as (temporary) protections against waves, modifying shipping routes, setting speed limits or limiting ship traffic to periods where the tidal water level lies lower than the restoration area. These measures will have to be adapted to site-specific conditions.
